# Regulation of the encapsulation, protection, and controlled release properties of naringin by sodium alginate oxidized hydrogel as a steady-state delivery system

**DOI:** 10.3389/fnut.2026.1809564

**Published:** 2026-03-30

**Authors:** Shuxian Bai, Lei Chen, Hui Teng, Hesham R. El-Seedi, Junyan Yu, Chao Ai

**Affiliations:** 1 Guangdong Provincial Key Laboratory of Aquatic Product Processing and Safety, Guangdong Province Engineering Laboratory for Marine Biological Products, Guangdong Provincial Engineering Technology Research Center of Seafood, Key Laboratory of Advanced Processing of Aquatic Product of Guangdong Higher Education Institution, College of Food Science and Technology, Guangdong Ocean University, Zhanjiang, China; 2 Shenzhen Institute of Guangdong Ocean University, Shenzhen, China; 3 International Research Center for Food Nutrition and Safety, Jiangsu University, Zhenjiang, China; 4 Department of Chemistry, Faculty of Science, Islamic University of Madinah, Madinah, Saudi Arabia

**Keywords:** bioactive compound delivery, encapsulation, hydrogel, naringin, sodium alginate

## Abstract

**Introduction:**

Addressing the application challenges of low oral bioavailability and poor chemical stability associated with the citrus flavonoid naringin, hydrogels featuring unique three-dimensional network structures have garnered significant attention in the field of bioactive compound delivery.

**Methods:**

In this study, a natural polysaccharide hydrogel delivery system based on dynamic imine bonds was designed and constructed. By regulating the oxidation degree of oxidized sodium alginate (OSA), hydrogels with distinct pore sizes were engineered to achieve stable encapsulation and controlled release of naringin. Dynamic covalent crosslinking between the aldehyde groups on OSA chains and the amino groups of carboxymethyl chitosan (CMC) endowed the hydrogels with exceptional adaptability.

**Results:**

Research indicates that increasing the oxidation state of OSA can form a denser cross-linked network, thereby significantly regulating the swelling behavior, biodegradation rate, and drug release kinetics of hydrogels. Among these, 6OSA-Hy exhibits the most ideal sustained-release properties, enabling continuous and controlled release of naringin in simulated environments. *In vitro* cell experiments confirmed that this delivery system exhibits excellent biocompatibility, and its released active components effectively promote cell migration, with the 6OSA-Hy group achieving a scratch closure rate of 43.5%.

**Discussion:**

The above studies demonstrate that the hydrogel system developed in this research provides a sustained-release stabilization strategy for enhancing the delivery of bioactive compounds such as naringin. This approach holds potential for improving bioavailability and is expected to find applications in the development of functional foods and nutritional supplements.

## 1 Introduction

Citrus bioflavonoids are primarily found in citrus fruits such as grapefruit, oranges, and lemons (1). Extensive research indicates that flavonoids exert positive effects on human health, including cancer prevention, cardiovascular disease prevention, antibacterial infection protection, anti-inflammatory properties, and neuroprotection. These benefits stem from their potent antioxidant activity (2, 3). Naringin is a citrus bioflavonoid extracted from citrus plants and is used to treat various diseases (4). Among natural citrus flavonoids, naringin has been evaluated as a potential anti-inflammatory and anticancer agent (5). However, due to the inherent poor water solubility of flavonoids, naringin exhibits low bioavailability and poor stability in aqueous solutions, and its functional applications are often limited. To effectively enhance the *in vivo* bioavailability of naringin, two key approaches are improving solubility and enhancing controlled-release effects (6).

Hydrogels are a novel class of functional polymeric materials featuring a three-dimensional network structure that mimics the architecture of the extracellular matrix (ECM). Due to their exceptional biocompatibility and functional properties, they have garnered significant attention in the field of active substance delivery (7). Based on raw material composition, hydrogels are broadly categorized as either synthetic or natural polymer-based. Natural polymer hydrogels generally offer greater application potential than their synthetic counterparts, as their biological origin affords them superior biocompatibility and biodegradability (8). Polysaccharides, which are carbohydrates polymerized from multiple monosaccharides, are widely found in animals, plants, and microorganisms and possess diverse physiological activities. Marine polysaccharides, such as chitosan, hyaluronic acid, and sodium alginate, are widely used in the field of wound dressings due to their excellent antibacterial, anti-inflammatory, and other biological activities (9).

Sodium alginate (SA) is a linear unbranched polysaccharide extracted from brown algal cell walls, consisting of varying ratios of 1,4′-linked *β*-D-mannuronic acid (M) and *α*-L-guluronic acid (G) residues. These residues are arranged in a block-like pattern along the chain, and differences in their composition and sequence are key to the diversity of its material properties (10). Owing to its excellent biocompatibility, biodegradability, and high moisture retention, SA is widely used in tissue engineering, and bioactive compound delivery (11).

**SCHEME 1:**
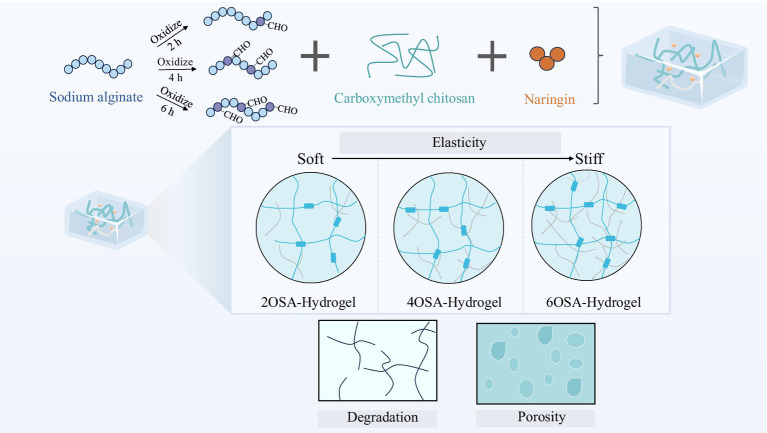
Schematic illustration of the preparation and physicochemical characterization of naringin-loaded oxidized sodium alginate and carboxymethyl chitosan hydrogels.

This study aims to develop a stabilized delivery system based on natural polysaccharides to address the stability and controlled release challenges faced by naringin as a bioactive nutrient. Through controlled oxidation of sodium alginate (SA), a series of oxidized sodium alginate (OSA) with varying degrees of oxidation was prepared. By utilizing the aldehyde groups generated *in situ* on the OSA chain to undergo a dynamic Schiff base reaction with the amino groups of carboxymethyl chitosan (CMC), we successfully constructed a hydrogel network exhibiting both excellent biocompatibility and self-healing properties. This network was employed as an encapsulation and delivery carrier for naringin (Scheme 1). Research indicates that this structural regulation directly governs the swelling behavior, biodegradation rate, and three-dimensional microporous structure of hydrogels. By modulating the oxidation state of OSA, the crosslinking density, hydrophilicity/hydrophobicity, and reversibility of dynamic bonds within the three-dimensional network structure can be precisely controlled. These physicochemical properties collectively provide the foundation for achieving controlled release of naringin. Therefore, this study successfully reports a sodium alginate-based hydrogel with outstanding performance. By regulating the structure of the carrier, we optimized its protective effect on naringin and its sustained-release properties. This provides both theoretical foundations and practical solutions for developing delivery systems tailored for functional foods and oral nutritional supplements.

## 2 Materials and methods

### 2.1 Materials

Sodium alginate (SA, AR), Carboxymethyl chitosan (CMC, Degree of substitution: ≥90%), and Naringin (Nar, Moligand™, ≥95%) were purchased from Aladdin Biochemical Technology Co. Ltd. (Shanghai, China). Sodium periodate (NaIO_4_, 99.5%), Ethylene glycol (EG, 98%), Hydroxylammonium chloride (H_3_NO·HCl, 98.5%), Methylene Blue Trihydrate (MB, Indicator) were purchased from Macklin biochemical technology Co., Ltd. (Shanghai, China). Dialysis bags (Mw = 3,500 Da) were purchased from Yuanye Biotechnology Co., Ltd. (Shanghai, China). CCK-8 assay kit was purchased from Abbkine Scientific Co., Ltd. (Wuhan, China).

### 2.2 Preparation of oxidized sodium alginate

Oxidized sodium alginate (OSA) was synthesized following a reported method (12) with slight modifications. Briefly, 10 g of sodium alginate was dissolved in 50 mL of anhydrous ethanol to form a suspension. A solution of 8 g sodium periodate in 50 mL deionized water was then added to this suspension. The reaction mixture was magnetically stirred for 2, 4, or 6 h at room temperature under light protection. To terminate the reaction, 3 mL of ethylene glycol was added and stirring was continued for 1 h. The product was purified by adding a large excess of anhydrous ethanol under vigorous stirring to induce precipitation. The precipitate was collected by vacuum filtration, and this purification step was repeated twice. The resulting white solid was redissolved in deionized water and rotary-evaporated at 60 °C to remove residual ethanol. The products were dialyzed using deionized water for 24 h (retention MW = 3,500 Da) with water changes every 12 h to remove unreacted sodium periodate and small molecule impurities such as ethylene glycol. The liquid inside the dialysis bag was freeze-dried for 48 h to obtain sodium alginate 2OSA, 4OSA, and 6OSA with different oxidation degrees.

To determine the degree of oxidation of sodium alginate (OSA), 0.1 g of the substance was added to 25 mL of a 0.25 mol/L hydroxylamine hydrochloride solution. The mixture was then stirred uniformly for 2 h using a magnetic stirrer. Titration was then performed using 0.1 mol/L NaOH to determine the concentration of HCl released from the solution. Measurements were taken using a pH meter, and titration was stopped when the solution’s pH stabilized at 5.0. Oxidation Degree (OD) was calculated as equation (1) according to the previous study (13):


$$\mathrm{OD}(\%)=\frac{\left(△V\times 0.001\times C_{\text{NaOH}}\right)}{m/198.11}\times \frac{1}{2}\times 100\%$$ (1)

where ΔV is the volume of NaOH solution consumed (mL), C_NaOH_ is the molar concentration of NaOH (mol/L), m is the mass of OSA (g), and 198.11 is the average molecular weight of the sodium alginate repeating unit (g/mol).

### 2.3 Fourier infrared spectroscopy

Sodium alginate (SA) and sodium alginate with different oxidizations (2OSA, 4OSA, 6OSA) were used as samples to be tested, pressed using dried potassium bromide and measured by infrared spectroscopy using a BRUKER TENSOR 27 Fourier infrared spectrometer in the range of 4,000–400 cm^−1^.

### 2.4 Preparation of naringin-loaded hydrogel

Oxidized sodium alginate solution at a concentration of 4 mg/mL was prepared by dissolving OSA of different oxidation degrees in deionized water and stirring magnetically for 30 min. CMC was dissolved in deionized water and magnetically stirred for 2 h to obtain carboxymethyl chitosan solution at a concentration of 4 mg/mL. A 1:1 mixture of carboxymethyl chitosan solution and various oxidized sodium alginate solutions was prepared, to which a 10% naringin solution was added to form a mixed precursor solution. After allowing the precursor solution to stand for 10 min, naringin-loaded hydrogels were obtained and designated as: 2OSA-Hydrogel, 4OSA-Hydrogel, and 6OSA-Hydrogel.

### 2.5 Microstructure

The hydrogel microstructure was observed by a Thermo Fisher Apreo 2C scanning electron microscope (SEM), and the 2OSA-Hydrogel, 4OSA-Hydrogel, and 6OSA-Hydrogel hydrogels were treated by freeze-drying, quenched and cooled by liquid nitrogen and then plated with gold in order to evaluate the three-dimensional stereo morphology and porosity of the hydrogels.

### 2.6 Self-healing properties

Two identical circular hydrogels were prepared, one of which was stained using methylene blue so that the unstained and stained gels carried different colors. Two round hydrogels were cut into two semicircles using a razor blade and different colored semicircular hydrogels were placed tightly together to make them self-healing (14). Photographs were taken at different time points to record the healing process, and the healing hydrogel was lifted up by hand during different time periods, and did not fall off by gravity and cut out without rupture by adhesive pulling to prove the success of self-healing.

### 2.7 Rheological property

Hydrogels of size 1 cm × 1 cm were placed on parallel plates with a gap of 1 mm and frequency scanning tests were carried out using a HAAKE MARS60 rheometer in an oscillating mode at 25 °C with a strain amplitude of 2% in the frequency range of 0.1–10 Hz to determine the storage modulus (G’) and the loss modulus (G”) (15). Next, temperature tests were performed at a strain amplitude of 2%, with the temperature increasing from 25 °C to 70 °C, to evaluate the changes in G’ and G.”

### 2.8 TPA

Referring to Liu method (16), hydrogel blocks of uniform thickness of 1 cm × 1 cm in size were placed on parallel plates and texture tests were performed using TA. XT plus C type texture meter at a test speed of 1 mm/s at 20% strain and 5 g trigger force to determine the hardness, adhesiveness, springiness and cohesiveness of the naringin-loaded hydrogels.

### 2.9 Adhesion test

Apply the naringin-loaded hydrogels to the back of the hand and bend the fingers to observe whether it adheres tightly or slips off. Simultaneously, place the hydrogel on a bent knuckle and then invert the finger or twist the joint to observe whether it remains firmly adhered.

### 2.10 Dissolution and degradation properties

The naringin-loaded hydrogels were weighed and immersed in pH 7.2–7.4 PBS and placed at 37 °C. At the indicated time points, the excess PBS on the surface of the hydrogel was wrapped from the hydrogel with absorbent paper and weighed again. The swelling ratio was calculated as equation (2) according to the previous study (17):


$$\text{Swelling ratio}\;(\%)=\frac{\left(W_{1}-W_{0}\right)}{W_{0}}\times 100\%$$ (2)

where W_0_ represents the initial hydrogel mass and W_1_ represents the swelled hydrogel mass.

The freeze-dried naringin-loaded hydrogels were weighed and immersed in pH 7.2–7.4 PBS and placed at 37 °C. At the indicated time points, the hydrogels were removed and excess PBS was removed from the surface of the hydrogels with absorbent paper and weighed after 48 h of freeze-drying. The degradation rate was calculated as equation (3) according to the previous study (17):


$$\text{The degradation rate}\;(\%)=\frac{\left(W_{0}-W_{1}\right)}{W_{0}}\times 100\%$$ (3)

where W_0_ represents the mass amount of the initial hydrogel and W_1_ represents the mass of the degraded hydrogel.

### 2.11 *In vitro* release studies

The cumulative release of naringin (NAR) was determined by Cary 60 UV–visible spectrophotometer, and the UV absorption spectra of different concentrations of naringin solutions were measured by using PBS as the solvent, and the absorbance values corresponding to the maximal peak of 284 nm were fitted to the standard curve.

The naringin-loaded hydrogels (2OSA-Hydrogel, 4OSA-Hydrogel and 6OSA-Hydrogel) samples were cut into sizes of about 1 g into dialysis bags and placed in PBS, and then incubated in a thermostatic shaking chamber at 37 °C with an oscillation rate of 100 rpm; 3 mL of the sample solution was taken out at regular intervals and the absorbance was measured at its maximum absorption peak at 284 nm, and an equal volume of fresh PBS was added, and the cumulative release of naringenin was calculated from the standard curve and the measured absorbance values at different time intervals (18).

### 2.12 Hemolysis

Fresh rat blood was taken and centrifuged (2000 rpm) for 10 min and the supernatant was discarded, the supernatant was washed with PBS (pH 7.2–7.4) and clarified to obtain erythrocytes, which were diluted with PBS to obtain a 4% erythrocyte suspension. The naringin-loaded hydrogels were co-incubated with the erythrocyte suspension for 2 h at 37 °C. The solution was centrifuged at 2000 rpm for 10 min and the absorbance was measured at 540 nm. 0.2% Triton X-100 and PBS were used as positive and negative groups, respectively (19).

The hemolysis ratio was quantified by the following equation (4):


$$\text{Hemolysis ratio}\;(\%)=\frac{A_{s}-A_{n}}{A_{p}-A_{n}}\times 100\%$$ (4)

where As denotes the absorbance value of the experimental group, Ap denotes the absorbance value of the positive control group and An denotes the absorbance value of the negative control group.

### 2.13 *In vitro* biocompatibility test

Mouse fibroblasts (L929) were inoculated in 96-well plates at 1 × 10^4^ cells/well and incubated at 37 °C and 5% CO_2_ for 24 h. After the cells adhered, the fresh medium containing different concentrations of hydrogel was replaced, and after an interval of 24 h, the medium was removed and replaced with 10% CCK-8 reagent solution. After incubation for 30 min, cell competence was assessed by measuring absorbance at 450 nm using an enzyme marker to determine the concentration of naringin-loaded hydrogels administration.

L929 cells were inoculated in 96-well plates and replaced with 10 mg/mL fresh medium containing naringin-loaded hydrogels after cell adhesion. After 24 h and 72 h intervals, the medium was removed and replaced with 10% CCK-8 reagent solution. After incubation for 30 min, cell proliferative capacity was assessed by measuring the absorbance at 450 nm using an enzyme marker. Live/dead staining of L929 cells was also assessed with Calcein-AM/Propidium Iodide Dye. Fluorescence images of cell morphology were observed using an inverted fluorescence microscope (20).

### 2.14 Cell migration

To explore the cell migration behavior of naringin-loaded hydrogels-treated L929 cells, a cell scratch assay was used. L929 cells were inoculated in 6-well plates at 4 × 10^5^ cells/well and cultured until 70–80% confluence was reached, and scratches of the same width were formed in each well by a 10 μL pipette tip. Subsequently, naringin-loaded hydrogels were added to co-incubate with the cells for 24 h and cell migration images were taken at 12-h intervals. The scratch closure rate was calculated as equation (5) according to the previous study (21):


$$\text{Scratch closure ratio}\;(\%)=\frac{\left(S_{i}-S_{t}\right)}{S_{i}}\times 100\%$$ (5)

where S_i_ and S_t_ denote the initial scratched area and scratched area at different time points, respectively.

### 2.15 Data analysis

This study used GraphPad Prism 8.0 software for data visualization and significance analysis. Graphs were plotted using Origin software (2021). Results are expressed as mean ± standard deviation of at least three experimental replicates. Statistical significance between samples was analyzed using one-way ANOVA (analysis of variance, multiple groups). Statistically significant differences (*p*-value) < 0.05 were considered statistically significant.

## 3 Results and discussion

### 3.1 Synthesis of OSA and its infrared structure

**Figure 1:**
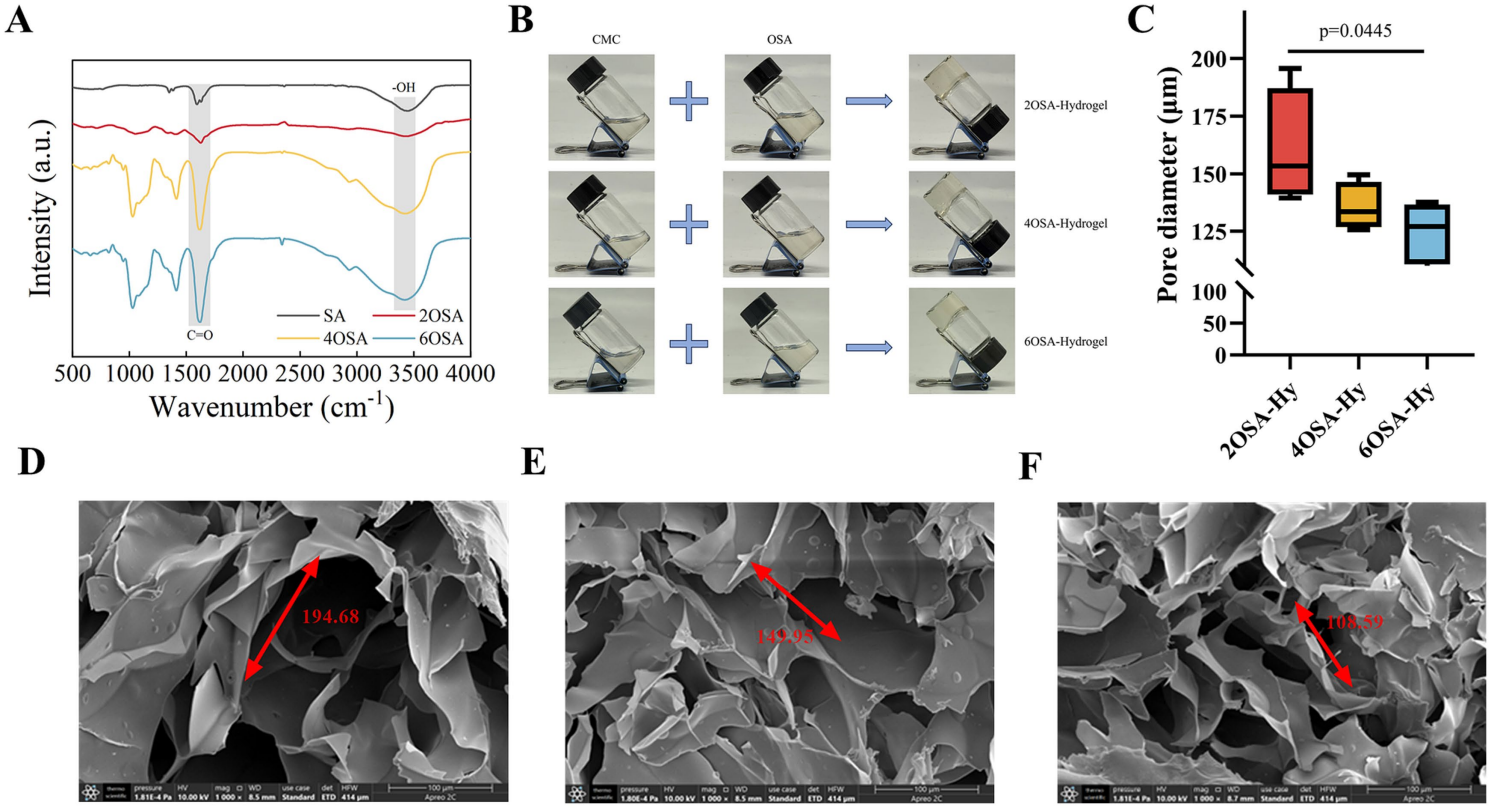
Microstructure and self-healing properties of naringin-loaded hydrogels. **(A)** Infrared spectra of sodium alginate with different degrees of oxidation. **(B)** Photographs of naringin-loaded hydrogels preparation process. **(C)** Pore diameter of Naringin-loaded hydrogels with different oxidation degree. SEM images of **(D)** 2OSA-Hydrogel, **(E)** 4OSA-Hydrogel, and **(F)** 6OSA-Hydrogel.

The degree of oxidation of sodium alginate determines the aldehyde group content on its molecular chain, serving as a key factor in regulating the density of the dynamic imine cross-linking network and closely related to its capacity for loading naringin. In order to investigate the degree of oxidation of sodium alginate, NaOH was used for titration, and the results of the consumed volume and the degree of oxidation are shown in Table 1, while the results show that the degree of oxidation of sodium alginate increases significantly with the prolongation of the action time of sodium periodate. This is due to the sodium periodate as a strong oxidant can make the sodium alginate molecular chain C-C bond fracture, at the same time in the fracture of the C_2_, C_3_ position to generate new aldehyde group, for example, 2OSA oxidation degree of about 61%. The oxidation degree of sodium alginate increased slowly at the later stage, 69 and 75% for 4OSA and 6OSA, respectively, which may be attributed to the fact that with the prolongation of the oxidation reaction time, the fracture sites on the molecular chain of sodium alginate were gradually reduced, and accompanied by the continuous consumption of sodium periodate, the reaction rate gradually slowed down and reached the equilibrium. To further confirm the degree of oxidation of sodium alginate at different reaction times, its molecular structure was characterized and the results are shown in Figure 1A. In the FTIR spectrograms, SA and OSA showed similar spectral profiles, with broad diffraction peaks in the range of 3,700–3,000 cm^−1^, which were generated due to the characteristic peak vibration of -OH. The characteristic peak at around 1700 cm^−1^ is due to the -C=O vibration, and it is noteworthy that OSA shows a significant enhancement of the characteristic peak attributed to the C-O absorption vibration peak of the aldehyde group compared to SA (22). And the stronger -C=O vibration peaks were observed with increasing reaction time, which further proved that more aldehyde groups were generated, i.e., sodium alginate was oxidized to a higher extent.

**Table 1:** Oxidation degree and NaOH consumption volume of sodium alginate at different treatment times.

Sample	△V_1_ (mL)	△V_2_ (mL)	△V_3_ (mL)	Oxidation (%)
2OSA	6	6.2	6.4	61.41 ± 1.98
4OSA	6.9	7.1	7	69.34 ± 0.99
6OSA	7.3	7.8	7.5	74.62 ± 2.49

Use titration with NaOH to determine the degree of oxidation of sodium alginate.

### 3.2 Preparation of naringin-loaded hydrogels and microstructure

The formation process of naringin-loaded hydrogels with different oxidation degrees is shown in Figure 1B, and the crosslinking of OSA and CMC with different oxidation degrees can prepare stable naringin-loaded hydrogels, and through the Schiff base reaction, the aldehyde group of OSA generates a dynamic imine bond with the amino group on CMC, which endows the naringin-loaded hydrogels with excellent in-situ gelation properties (23). In order to investigate whether there are differences in their microstructures, 2OSA-Hy, 4OSA-Hy and 6OSA-Hy were observed by scanning electron microscopy. In order to investigate whether there are differences in their microstructures, 2OSA-Hy, 4OSA-Hy and 6OSA-Hy were observed by scanning electron microscopy. The denser degree of cross-linking determines the porosity of the hydrogel, as shown in Figure 1C; the pore size of 6OSA-Hy (124.8 μm) is lower than that of 2OSA-Hy (160.5 μm) and 4OSA-Hy (135.6 μm). The SEM images of 2OSA-Hy, 4OSA-Hy and 6OSA-Hy are shown in Figures 1D–F. The results showed that there were differences in the degree of cross-linking and the uniformity of the network formed by the naringin-loaded hydrogels. With the increase of oxidation degree, the naringin-loaded hydrogels showed a trend of denser cross-linking and smaller pore size. It is speculated that this should be due to the fact that the higher the oxidation degree of OSA and the more aldehyde groups, the more cross-linking points can be formed by reacting with the amino groups of CMC to form a uniformly distributed and dense three-dimensional network. Simultaneously, the densely crosslinked network structure with smaller pores effectively restricts the outward diffusion of naringin after encapsulation, thereby enhancing its encapsulation efficiency.

### 3.3 Self-healing properties of naringin-loaded hydrogels

**Figure 2:**
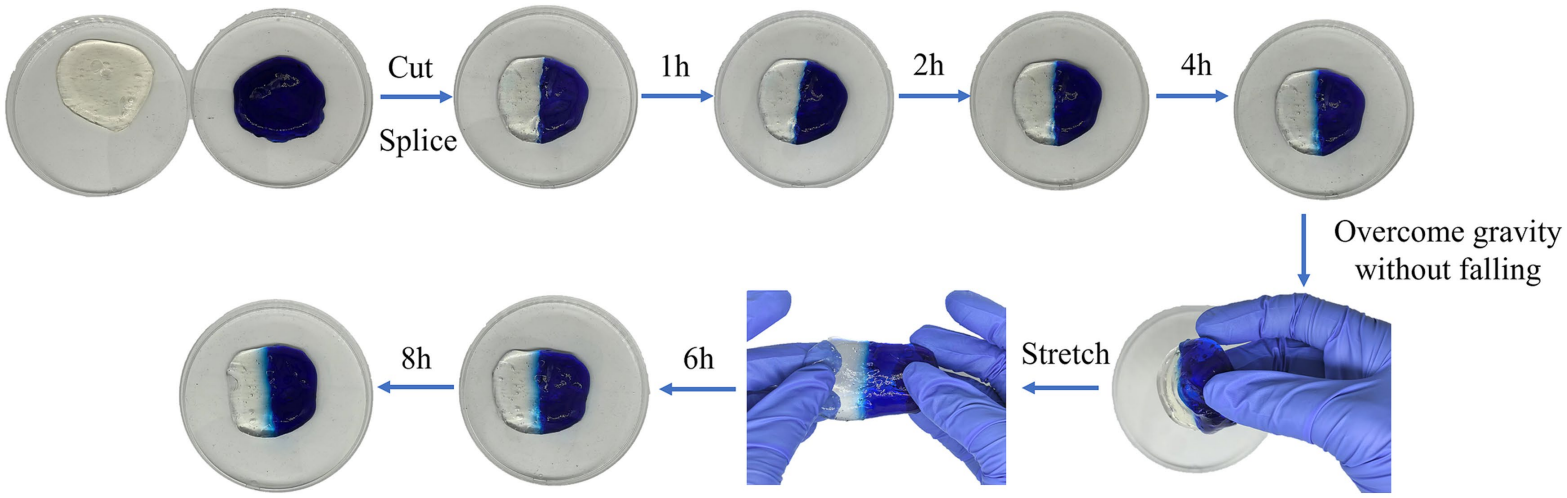
Demonstration of the self-healing property of the naringin-loaded hydrogel.

The dynamic imine bonds generated through Schiff base reactions can be utilized to construct dynamic cross-linked networks, which offer unique advantages for encapsulating, protecting, and delivering bioactive molecules such as naringin. To further verify the reversible dynamic imine bonding produced by the Schiff base reaction, the naringin-loaded hydrogel was made into a circular hydrogel using methylene blue staining, cut and re-spliced onto the unstained semicircular hydrogel. The results are shown in Figure 2. After 4 h of reintegration and splicing, the reassembled hydrogel has been completely healed into the integrated original circular shape with no damage or crack lines (20). The reconstituted hydrogel was not dropped by gravity, and the splices of the hydrogel did not fracture and fall off after strenuous pulling, and could still recover the original shape, and the dynamic imine bond was still unbroken and the dye molecules continued to diffuse. This demonstrated that the oxidatively modified sodium alginate could cross-link with carboxymethyl chitosan to produce reversible dynamic imine bonds, which showed excellent self-healing properties. This enabled the hydrogel to restore its crosslinked structure after external compression, ensuring a more sustained and stable naringin delivery system.

### 3.4 Mechanical properties of naringin-loaded hydrogels

**Figure 3:**
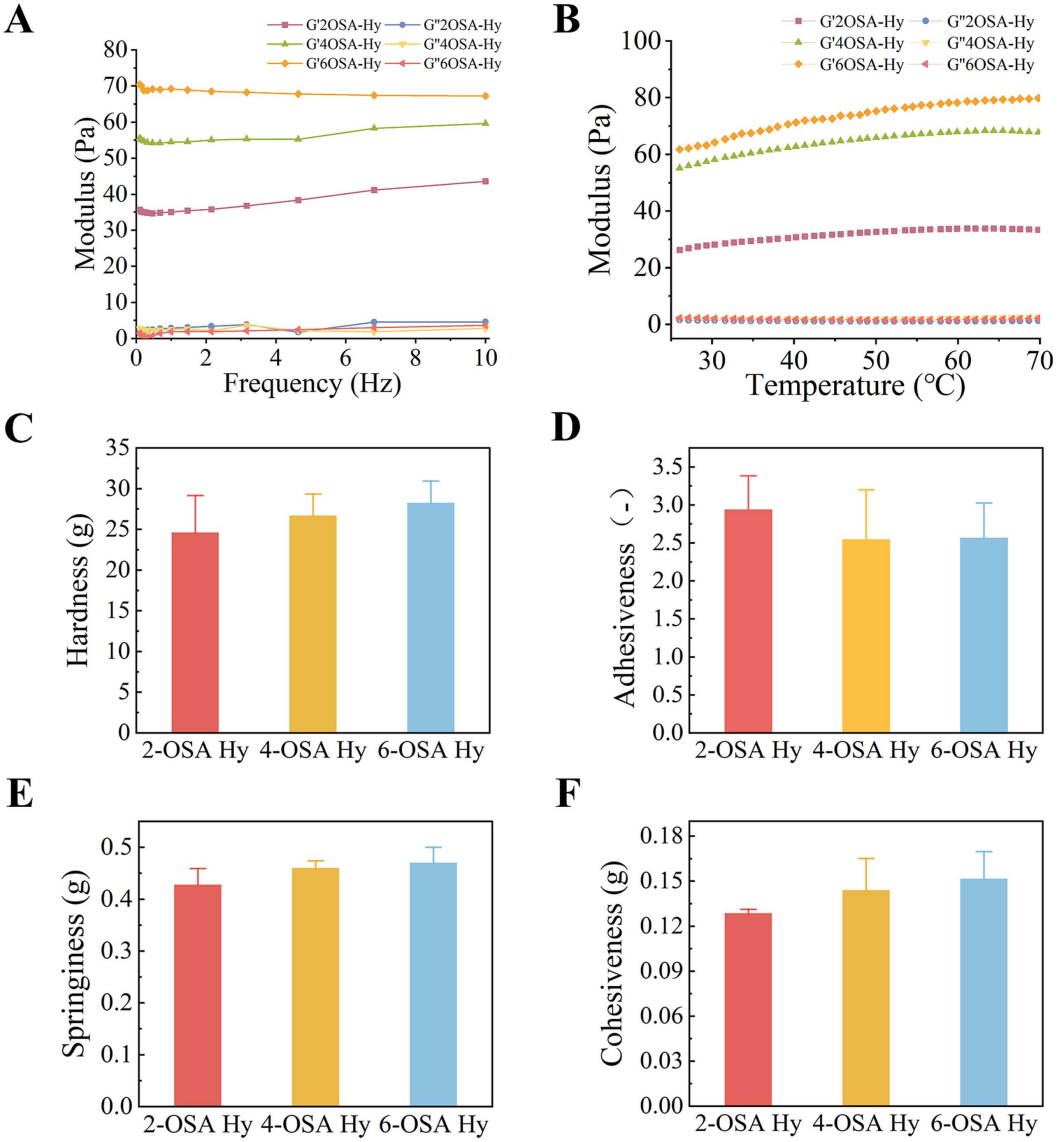
Physical characterization of naringin-loaded hydrogels with different degrees of oxidation. Curves of storage modulus G’ and loss modulus G” versus **(A)** frequency and **(B)** temperature for naringin-loaded hydrogels with different degrees of oxidation. **(C)** Hardness, **(D)** adhesiveness, **(E)** springiness, **(F)** cohesiveness of naringin-loaded hydrogels with different degrees of oxidation.

Rheodynamic studies link the microstructure of hydrogels to their macroscopic properties to investigate the effects of OSA with varying degrees of oxidation on naringin-loaded hydrogels formation, the rheological behavior of naringin-loaded hydrogels over time was analyzed by oscillating frequency scanning (0.1–10 rad/s) experiments. As shown in Figure 3A, the elastic modulus (G’) is always higher than the loss modulus (G”), indicating that all naringin-loaded hydrogels have a stable structure still retaining the solid state, always dominated by the elastic behavior. The elastic modulus (G’) of naringin-loaded hydrogels with different degrees of oxidation 6OSA-Hy > 4OSA-Hy > 2OSA-Hy demonstrates that with the increase of aldehyde groups attached to OSA, the more densely crosslinked the hydrogel, the higher the elastic modulus, i.e., the more elastic it is. Meanwhile, the rheological behavior of naringin-loaded hydrogels over time was analyzed by temperature–frequency scanning experiments (Figure 3B), and the hydrogels with different oxidations from 25 °C to 70 °C always maintained the elastic behavior, and there was no obvious critical temperature point of phase transition in the hydrogels. These results indicate that the phase change process of hydrogels occurs gradually. This phenomenon can be attributed to the high concentration of polymers producing a high degree of molecular entanglement and cross-linking in the sol–gel state, which is more inclined to exhibit the elastic modulus of elastic materials higher than the viscous modulus (24), further authenticating the good stability of the hydrogel structure.

During actual processing, hydrogels undergo deformation due to mechanical stress, leading to reduced stability of the encapsulated naringin. Further investigation and optimization of the mechanical properties of naringin-loaded hydrogels are therefore critical. Good mechanical strength ensures that hydrogels are not extruded and deformed during use (25). To assess the mechanical properties of hydrogels, compression testing of hydrogel samples was carried out using a TPA. The hardness, elasticity, adhesion, and cohesion of hydrogels were shown in Figures 3C–F. The hardness, elasticity, and cohesion increased with the increase of OSA oxidation, and the adhesion decreased with the increase of OSA oxidation. This was consistent with the rheological results of naringin-loaded hydrogels. The increase in hardness and elasticity of 6OSA-Hy likely stems from greater entanglement between naringin molecules and polysaccharide chained after encapsulation, thereby enhancing the mechanical properties of naringin-loaded hydrogels. Among them, hardness directly reflected the stiffness and crosslink density of the hydrogel polymeric network, and higher hardness represents a firmer hydrogel. The elasticity was measured as the height of recovery of the hydrogel within the waiting time after the first compression, indicating that the denser the degree of crosslinking of the hydrogel the better its recovery performance and the better its elasticity. Cohesion is the ability of hydrogel to keep its internal structure intact after the first compression deformation. It also directly reflected the strength of the internal network structure of the hydrogel, and a densely cross-linked network was able to resist damage, so the cohesion was high. The adhesion of naringin-loaded hydrogels decreased with increasing concentration, as adhesion was primarily influenced by intramolecular friction between naringin and the polymer macromolecules, whereas TPA did not alter the structure. Therefore, hydrogels with highly crosslinked structures resulting from higher oxidation levels may increase system friction, thereby affecting adhesion (16).

**Figure 4:**
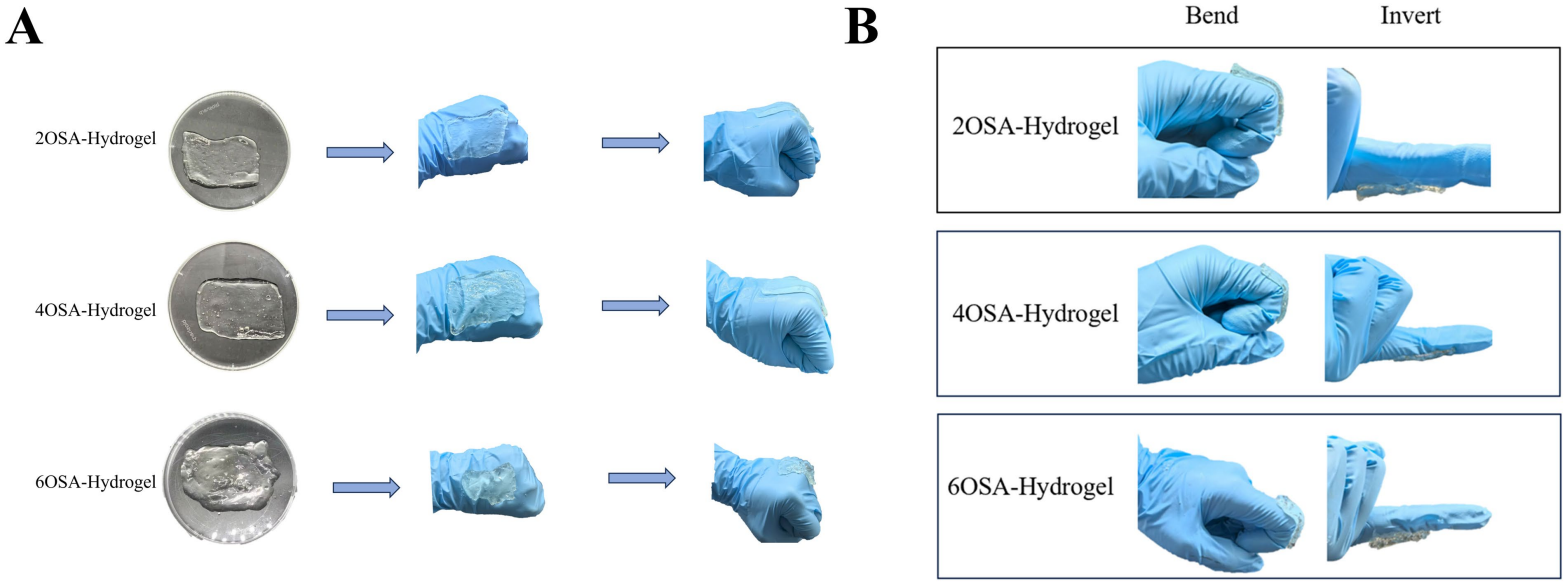
Adhesive properties of naringin-loaded hydrogels: **(A)** adhesion test on the back of a bent hand and **(B)** adhesion test by bending and inverting the finger.

Prior to conducting bioavailability studies on naringin, these novel delivery systems were tested using human dorsal hand skin. In practical applications, imparting adhesive properties to hydrogels is crucial for enhancing their reliability and stability (26). To ensure the gel maintains long-term stability in protecting naringin, hydrogels with varying degrees of oxidation were applied to the back of the hand and the flexed finger joints for testing. The results are shown in Figures 4A,B. The introduction of carboxymethyl chitosan, with its abundant carboxyl and amino groups forming strong bonds, combined with the dynamic covalent cross-linking generated by the Schiff base reaction, collectively endows the hydrogel with exceptional adhesive properties. This enables it to firmly adhere to the back of the hand and active joints (27). These results demonstrate that naringin-loaded hydrogels exhibit both toughness and self-healing capabilities, along with strong tissue adhesion. They can rapidly restore structural integrity under dynamic physiological conditions, thereby effectively preserving the bioactivity of naringin (28).

### 3.5 Swelling properties, degradation behavior, and *in vitro* release of naringin-loaded hydrogels

**Figure 5:**
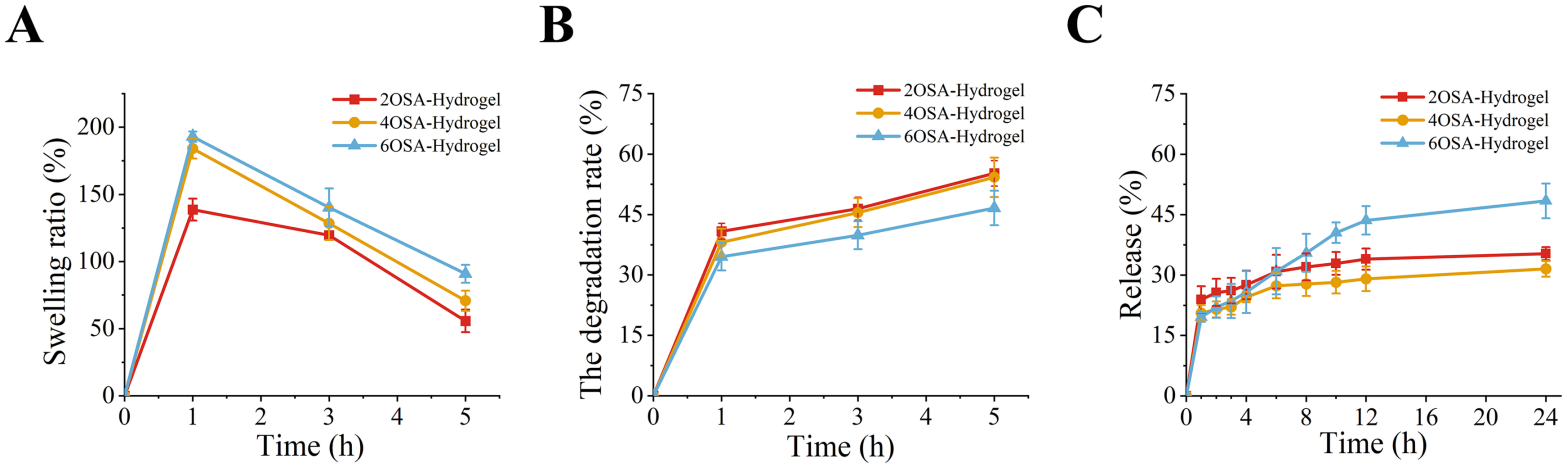
Characterization of physicochemical properties of naringin-loaded hydrogels with different degrees of oxidation: **(A)** swelling, **(B)** degradation, and **(C)** release properties of hydrogels.

Hydrogels exhibit the ability to absorb and retain foreign water molecules due to the three-dimensional network structure they possess and the hydrophilic properties unique to sodium alginate (17). The swelling ability of naringin-loaded hydrogels was tested and the results are shown in Figure 5A. In the first 1 h, the swelling rate of naringin-loaded hydrogels with different oxidation degree increased very much, and with the extension of the soaking time of naringin-loaded hydrogels in buffer solution, the swelling rate decreased slowly, which may be due to the role of a large number of hydrophilic groups on the polar molecular chain of sodium alginate degraded gradually and slowly. Simultaneously, the results indicate that sodium alginate-based naringin-loaded hydrogels with higher oxidation levels exhibit greater swelling rates. This phenomenon was likely attributed to their densely crosslinked structure and enhanced porosity, which conferred superior water retention capacity and swelling properties. Consequently, these hydrogels more effectively prevented the diffusion of naringin molecules outward from the hydrogel matrix.

To achieve sustained release of naringin, the unique network structure of the hydrogel gradually dissociates, thereby meeting this requirement. The encapsulated naringin is continuously released as the matrix degrades, maximizing bioavailability (29). In the swelling performance test, we found that the mass of the naringin-loaded hydrogels began to decrease after incubation time of more than 1 h. Therefore, the naringin-loaded hydrogels will be lyophilized and processed and weighed at the 1, 3 and 5 h incubation time points in the swelling test. In Figure 5B it was found where 2OSA-Hy degraded 55.2% ± 3.1%, 2OSA-Hy degraded 54.2% ± 4.8% and 6OSA-Hy degraded 46.6% ± 4.2% after 5 h of incubation. The difference in the degradation rates of naringin-loaded hydrogels with different oxidation degrees was not significant, which may be due to the fact that the naringin-loaded hydrogels contained the same concentration of sodium alginate, while their inherent hydrophilic nature was not altered by oxidative modification. However, 6OSA-Hy, as the naringin-loaded hydrogel with the densest cross-linking structure, good mechanical properties and the smallest porosity, still has a low degradation rate, which proves that the degradation rate of naringin-loaded hydrogel is still closely related to its cross-linking density and mechanical properties.

The network barrier of hydrogels impedes the release of active substances (30), in view of this, the bioactive compound release behavior of hydrogels was evaluated. The results are shown in Figure 5C, naringin release rate increased dramatically within the first 1 h. This rapid release was attributed to the greater surface area interaction of the loaded naringenin in contact with the buffer, which leads to the release of the bioactive compound from the naringin-loaded hydrogel on the surface or in contact with the surface due to the large concentration difference. The rate of release also increased gradually between 2 and 5 h. It was surmised from the pre-swelling and degradation tests that it should be the beginning of degradation of the naringin-loaded hydrogel that led to the accelerated release. While between 6 and 12 h, the release rate started to slow down, a phenomenon that could be attributed to the loss of porosity and most of the naringin-loaded hydrogel degradation (31); 12–24 h the release rate became very slow and gradually tended to parallel, which could be related to the depletion of the release driving force with less naringin remaining in the structure. During the whole release process, we can find that the 6OSA-Hy produced by higher oxidation degree was released slowly in the early stage, but was released steadily and slowly in the late stage, and the highest release rate reached about 48.4% at 24 h. Due to its dense network structure hindering the rapid release of naringin internally, this sustained-release property could help maintain stable local bioactive compound concentrations, thereby enabling steady-state delivery of naringin.

### 3.6 *In vitro* biocompatibility of naringin-loaded hydrogels with cells

**Figure 6:**
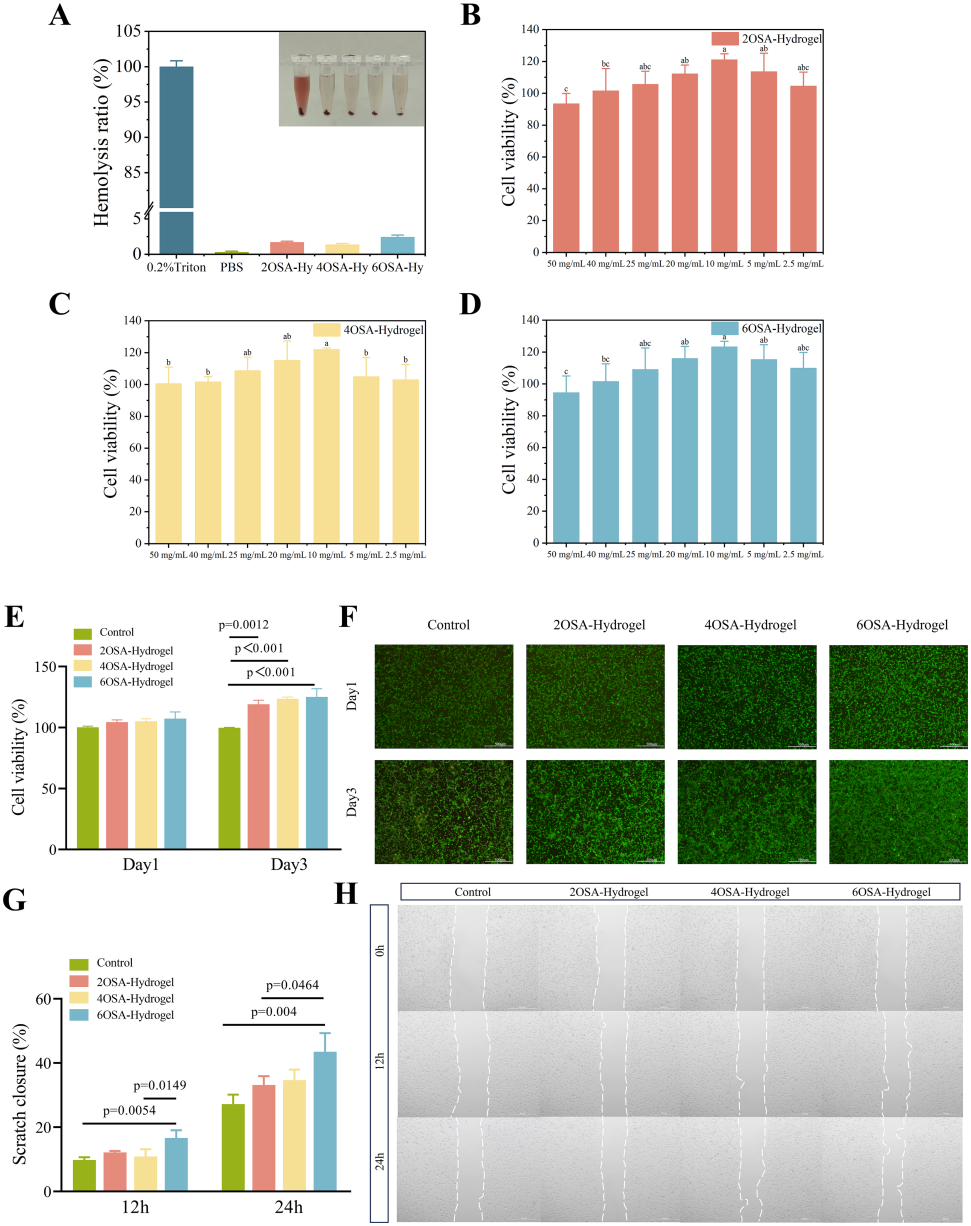
Evaluation of cytotoxicity and biocompatibility of naringin-loaded hydrogels with different degrees of oxidation. **(A)** Hemolysis rate of naringin-loaded hydrogels and representative pictures. Viability of L929 cells after treatment with different concentrations of **(B)** 2OSA-hydrogel, **(C)** 4OSA-hydrogel, and **(D)** 6OSA-hydrogel. Viability of **(E)** L929 cells after co-culture with hydrogels of different oxidative degrees for 24 h, 72 h, and **(F)** corresponding live/dead staining images of L929 cells. Quantitative results of L929 cell scratch **(G)** healing area assay and **(H)** representative images after treatment with naringin-loaded hydrogels of different oxidative degrees for 0, 12, and 24 h.

Excellent biocompatibility is the primary prerequisite for ensuring bioavailability. If a hydrogel interacts with biological tissues without causing adverse reactions, it demonstrates biocompatibility (32). To determine whether the naringin-loaded hydrogel can be utilized *in vivo*, a blood compatibility test was conducted, using PBS and 0.2% Triton X-100 solvent as negative and positive controls, respectively. The results was shown in Figure 6A, in contrast to the hemolysis occurred in 0.2% TritonX-100 group, the supernatants of PBS and all naringin-loaded hydrogels groups were clear in color, proving that there were fewer ruptured erythrocytes in all sample groups (19). And the hemolysis rate was less than 5%, where the results of hemolysis rate reflected the degree of damage to the erythrocyte material, and a high rate of hemolysis indicated that the erythrocytes were severely damaged (33). This demonstrates that naringin-loaded hydrogels with varying degrees of oxidation exhibit excellent biocompatibility, indicating their potential as delivery systems for bioactive substances within the body.

In order to further verify the biocompatibility of hydrogels, L929 fibroblasts were selected to verify the biocompatibility of naringin-loaded hydrogels with different oxidation degrees. The results of the concentration gradient experiments as Figures 6B–D showed that the cell viability peaked under the hydrogel extract containing naringin treatment of 10 mg/mL, indicating that the naringin-loaded hydrogels with different oxidative degree at this concentration had the strongest promotion effect on cell proliferation, and thus this concentration was selected for cell culture in the follow-up. After incubating cells with naringin-loaded hydrogels for 24–72 h, CCK-8 assays revealed cell viability exceeding 100% (Figure 6E), suggesting this effect was associated with naringin’s potent antioxidant and anti-apoptotic activities. Concurrently, live/dead cell staining revealed no apparent apoptosis in any L929 cells (Figure 6F). Furthermore, all cells labeled with green fluorescence exhibited well-defined spindle morphology (20), indicating that naringin-loaded hydrogels with varying oxidation levels also demonstrate excellent biocompatibility with cells. This safety assurance supports their potential for further *in vivo* applications.

### 3.7 Cell scratch assay of naringin-loaded hydrogels

In the field of functional food development, achieving effective delivery of flavonoids such as naringin presents two interrelated challenges: ensuring their stability during gastrointestinal transport while also guaranteeing the effective exertion of their bioactivity at target sites. Previous studies have demonstrated that naringin effectively promotes the proliferation of endothelial progenitor cells and the formation of tubules (34). Given that cell migration is a critical step in the angiogenesis process (21), to evaluate whether naringin retains its antioxidant and pro-angiogenic potential after encapsulation and controlled release. Systematically evaluated the effects of naringin-loaded hydrogels with different degrees of oxidation on the loading capacity of naringin and its bioactive release, this study further employed a cell scratch assay to investigate the promotion of cell migration by naringin within the hydrogel delivery system. Quantitative analysis (Figure 6G) indicated that at 24 h post scratch, the wound closure area in the 6OSA-Hy group reached 43.5%, significantly higher than the control group (27.2%), the 2OSA-Hy group (33.2%), and the 4OSA-Hy group (34.7%). Results showed that compared with the control group, the hydrogel containing naringin significantly promoted L929 cell migration (Figure 6H). This indicated that the naringin loaded in 6OSA-Hy exhibits stronger pro-migration activity, likely due to the system’s higher naringin loading efficiency and more stable release performance, thereby more effectively maintaining naringin’s bioavailability and biological activity.

## 4 Conclusion

In summary, this study successfully constructed and characterized a dynamically crosslinked hydrogel capable of regulating the delivery behavior of naringin. To address the critical issues of low bioavailability and poor chemical stability in naringin, this system achieves effective encapsulation, protection, and controlled release of naringin by regulating the oxidation degree of sodium alginate and crosslinking it with carboxymethyl chitosan. Research confirms that 6OSA-Hy exhibits the most ideal sustained-release effect, enabling continuous and controlled release of naringin. Simultaneously, the naringin released from the hydrogel effectively promoted cell proliferation and migration (with wound closure reaching 43.5% in the 6OSA-Hy group), demonstrating that the key biological activity of naringin remains fully preserved after encapsulation and controlled release via this delivery system. These findings demonstrate that this study not only provides an effective stabilization strategy to enhance the bioavailability of naringin, but also holds promise as a functional oral formulation for improving intestinal barrier function or delivering systemic health benefits. Looking ahead, subsequent work will focus on developing orally ingestible hydrogel microspheres or granules. These formulations will be combined with more advanced *in vitro* digestion models and intestinal permeability studies to directly evaluate and validate their efficacy in enhancing the bioavailability of naringin.

## Data Availability

The original contributions presented in the study are included in the article/supplementary material, further inquiries can be directed to the corresponding author.
